# Descriptive study on subjective experience of genetic testing with respect to relationship, family planning and psychosocial wellbeing among women with lynch syndrome

**DOI:** 10.1186/s13053-021-00194-x

**Published:** 2021-09-14

**Authors:** Mari Kalamo, Johanna Mäenpää, Toni Seppälä, Jukka-Pekka Mecklin, Kirsi Pylvänäinen, Synnöve Staff

**Affiliations:** 1 Department of Gynecology and Obstetrics, Tampere University Hospital, Tampere, Finland; 2 Faculty of Medicine and Health Technology, Tampere University, Tampere, Finland; 3 Tampere University Hospital Cancer Center, Tampere University Hospital, Tampere, Finland; 4 Department of Gastrointestinal Surgery, Helsinki University Hospital, Helsinki, Finland; 5 Department of Surgery, Central Finland Central Hospital, Jyväskylä, Finland; 6 Faculty of Sports and Health Sciences, University of Jyväskylä, Jyväskylä, Finland; 7 Department of Education and Science, Central Finland Health Care District, Jyväskylä, Finland

**Keywords:** Lynch syndrome, Hereditary cancer, Testing, Relationships, Psychosocial wellbeing

## Abstract

**Background:**

Due to increased risk of endometrial and ovarian cancer, women belonging to known Lynch Syndrome (LS) families are recommended to undergo germline testing. Current practice in Finland is to offer counselling to women with pathogenic variant and advocate risk-reducing surgery (RRS) after completion of childbirth. The present study aimed to clarify the impacts of positive germline testing on family planning and reproductive decisions of these women, which are relatively unknown.

**Methods:**

Seventy-nine carriers of germline MMR gene pathogenic variant (*path_MMR)* were identified from the Finnish LS Registry as having genetic testing performed before the age of 45 years and not having undergone hysterectomy or oophorectomy. These women were sent a questionnaire concerning family planning, intimate relationships and psychosocial wellbeing.

**Results:**

Thirty-five women (44.3%) responded. Parity of *path_MMR* carriers (2.1) was slightly higher than parity among Finnish women in general (1.8). No significant differences were found between parity, number of induced abortions or sterilizations before and after genetic testing. Only minority of subjects reported any influence on family planning (20%) or negative impact on feminine self and body image (14%).

**Conclusions:**

The positive germline testing does not seem to have a major negative impact on family planning, intimate relationships or feminine self and body image. According to the open comments, counselling, supportive and empathic attitude of the professionals seem to have a significant impact on this. These results are a valuable addition to the counselling of LS women at reproductive age.

## Background

Women with a pathogenic MMR gene variant (*path_MMR*) associated with Lynch syndrome (LS) carry a 40–70% lifetime risk for endometrial cancer and a 7–15% lifetime risk for ovarian cancer [1–3]. For female carriers of *path_MMR*, international guidelines recommend an annual review with an clinician after age of 25 and, if certain symptoms e.g. abnormal bleeding occur, also gynecological referral [4]. At the time of the present study, the procedure in Finland was a gynecological surveillance including pelvic ultrasound examination and endometrial biopsy with one or 2 years interval, beginning at age of 35 [5]. However, with no clear evidence of survival benefit supporting the gynecological screening procedure [5], Finland has recently revised the national guidelines concerning the gynecological screening (FinGOG guidelines, accessed December 2019: https://gynekologiyhdistys.fi/pienryhmat/onkologia).

Soon after positive germline testing and counselling by a clinical geneticist, Finnish women with LS are offered a visit at a tertiary hospital with an expert gynecologist providing additional counselling and clinical gynecological and ultrasound examination. Thereafter, routine screening visits are not any more recommended to symptomless individuals. Visits, including gynecological ultrasound examination and endometrial biopsy, are suggested if dysfunctional bleeding occurs. However, risk-reducing hysterectomy, possibly with oophorectomy, is recommended when childbearing is complete or at the age of 50 years at the latest and carriers of *path_MMR* are usually invited to discuss the timing of RRS at the age of 40 years [5].

Finnish LS Registry (LSRFi) comprises all known families with LS-associated inherited MMR variant in Finland [6]. The germline testing of members of these families is mainly performed in early adulthood depending on the individual’s preference. Germline testing as such may be associated with psychological distress and anxiety [7]. Having inherited *path_MMR* predisposing to gynecological cancers may also have an impact on intimate relationship, family planning and psychosocial wellbeing. There is a relative lack of data available concerning these aspects with respect to any hereditary cancer syndrome [8, 9]. A few studies have been performed on patient-physician -relationship and effects of surveillance, the *path_MMR* carrier’s knowledge about the surveillance and decision-making concerning the prophylactic surgery in LS [10–14]. However, little is known about influence of positive germline testing on parity, age and timing of childbearing, induced abortions, sterilizations, intimate relationships, feminine self and body image. This information would be useful and valuable to professionals when counselling and communicating with young women diagnosed with LS-associated germline variant.

In the present study, we aimed to collect information and aspects from female carriers of *path_MMR* considering their subjective experience of positive germline testing with respect to relationship, family planning and psychosocial wellbeing.

## Methods

### Study subjects

The present study was performed at Tampere University Hospital (TAUH), Tampere, Finland. The study protocol was approved by TAUH Ethical Committee (January 2011) and an informed consent was obtained from all the study participants.

**Fig. 1:**
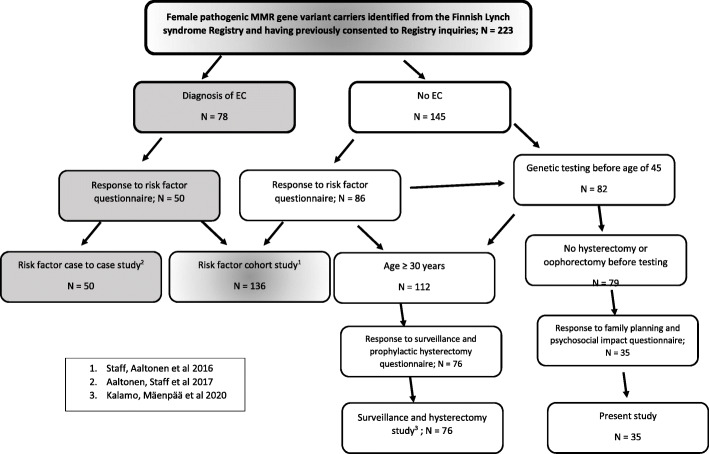
Study of gynecological health and morbidity among Finnish LS women

The present study is a part of a large retrospective cohort study among Finnish women with LS aiming at characterization of factors associated with gynecological health and morbidity in general [14, 15, 16]. The entire female LS study population, the present study and the previously published sub-studies are presented in a schematic flow chart (Fig. 1). The Finnish LS Registry (LSRFi) consists data of original research cohort including 81 kindreds ascertained through family history of LS and finally includes data of 1700 carriers of verified germline variant [6]. The women in the present study have given their informed consent to participate in LSRFi initiated clinical studies and permitted LSRFi researchers to use their address and medical information. They have all been voluntarily tested positive for MMR-gene pathogenic variant associated with Lynch syndrome, thus receiving appropriate information and counselling by the professionals.

The study population included women with pathogenic MMR gene variants identified from LSRFi and no history of endometrial cancer. Inclusion criteria for this study was germline testing before the age of 45 years (i.e. women considered at fertile age) and no hysterectomy or oophorectomy performed before germline testing (possibility to conceive after testing). Sterilization was not an exclusion criteria, as we consider it possible to wish for pregnancy and conceive through fertility treatments even after sterilization. Finally, 79 women were identified and a postal questionnaire concerning family planning and psychosocial effects of germline testing was sent to them. The questionnaire was re-sent within 6 months after first mailing to non-responding subjects. Demographics of the carriers included in the present study are presented in Table 1.

**Table 1:** Pathogenic MMR germline variant carriers included in the study

	Responded to questionnaire = study population (*N* = 35)	Non-responders (*N* = 44)	All (*N* = 79)
Median age at study	44 (31–59)	40 (24–56)	41 (24–59)
Median age at genetic testing	31 (21–42)	28 (19–44)	30 (19–44)
Hysterectomy performed after testing	6 (17.1%)	9 (20.4%)	15 (19.0%)
Parity (mean)	2.1 (0–4)	2.3 (0–5)	2.2 (0–5)
Gene:			
*MLH1*	28 (80,0%)	36 (81.8%)	64 (81.0%)
*MSH2*	4 (11.4%)	5 (11.3%)	9 (11.3%)
*MSH6*	3 (8.6%)	3 (6.8%)	6 (7.6%)

(*p* > 0,1 on all variables compared to responders)

### Questionnaire

The study questions included in the questionnaire are presented in Table 2. The questionnaire also included an opportunity to give open comments considering the effects of germline testing on family planning and further personal information about the topic.

**Table 2:** Questionnaire with given responses (*N* = 35)

Highest educational degree	High school	2 (5.7%)
	Vocational school	15 (42.9%)
	Uni. applied sciences	11 (31.4%)
	University	7 (20.0%)
Questions with answer options “yes” and “no”:		
1.Were you in a relationship at the time of testing?	Answer “yes”	32 (91.0%)
2. Were you in a relationship at the time of study?	Answer “yes”	28 (80.0%)
3. Did genetic testing have influence on your relationship?	Answer “yes”	1 (3.0%)
4. Have you been pregnant before testing?	Answer “yes”	24 (69.0%)
5. Have you been pregnant after testing?	Answer “yes”	17 (49.0%)
6. Have you had induced abortion(s) before testing?	Answer “yes”	2 (5.7%) (8.7% of women who answered “yes” to question 4.)
7. Have you had induced abortion(s) after testing?	Answer “yes”	2 (5.7%) (11.8% of women who answered “yes” to question 5.)*
8. Have you planned pregnancy before testing?	Answer “yes”	30 (86.0%)
9. Have you planned pregnancy after testing?	Answer “yes”	15 (42.9%)
10. Have you been sterilized before testing?	Answer “yes”	5 (14.3%)
11. Have you been sterilized after testing?	Answer “yes”	5 (14.3%)
12. Did genetic testing have influence on your family planning?	Answer “yes”	7 (20.0%)
13. Did genetic testing have negative influence on your feminine self and body image?	Answer “yes”	5 (14.3%)

*difference between the amount of abortions not statistically significant: *p* > 0,5

## Results

Finally, 35 women returned the questionnaire after two mailings, resulting in a 44.3% response rate. All of them reported attending the gynecological surveillance regularly.

Median age of the responders at study was 44 (31–59) years and their median age at germline testing was 31 (21–42) years. Median time interval between testing and the study was 13 (10–17) years. Mean parity of the responders was 2.1 (0–4). Mean parity of the non-responders was 2.3 (0–5) and mean parity of the whole study population was 2.2 (0–5).

The most common gene with MMR variant among the responders was *MLH1* (80.0%), reflecting the high percentage of *MLH1* carriers in LSRFi. The overall characteristics between the responders and non-responders were quite similar with no statistically significant differences. Details on responders as well as non-responders are summarized in Table 1.

All the responders had at least secondary vocational education. Twenty percent had a university degree. Most women reported being in a relationship at the time of genetic testing at a median age of 31 (91%), and practically all of them stated that testing had no influence on their relationships (97%). Before genetic testing, 86% of women had been pregnant and approximately half of the responders had also been pregnant after the testing. The number of reported induced abortions and sterilization procedures was similar before and after testing (5.7 vs 5.7% and 14.3 vs 14.3%, respectively). Only seven women experienced that positive germline testing influenced their family planning (20%). Only a small proportion of women reported negative impact on feminine self or body image (14%). Educational details and responses to questionnaire of the study responders are presented in Table 2.

Six out of 7 women who reported any impact of germline testing on family planning gave detailed information on this topic, and are presented in Table 3. Some women gave spontaneous, open descriptions of reactions related to germline testing and they are summarized in Table 4. Half of these comments included feelings of gratefulness and appreciation towards the testing procedure and health care professionals.

**Table 3:** Effects of genetic testing on family planning (Question 12 on Table 2: Reported by 7 women and 6 described the effects in more detail as abstracted here)

Age 34, tested at 27, 1 child before testing and 1 after testing, gene *MLH1*	Wanted to attend colonoscopy as planned and this had influence on pregnancy timing
Age 41, tested at 33, 1 child before testing and 3 children after testing, gene *MLH1*	Genetic finding limited the number of children, wanted to have them quickly after testing. Considered that pregnancies and breastfeeding have positive effects on health.
Age 39, tested at 21, 2 children after testing, gene *MLH1*	After genetic finding decided to have children as early as possible
Age 51, tested at 39, 2 children before testing, gene *MSH2*	Decided not to have more children after genetic finding.
Age 32, tested at 22, 1 child after testing, gene *MLH1*	Does not plan pregnancy after 35, thinks endometrial sampling affects fertility
Age 46, tested at 29, 1 child before testing, gene *MLH1*	After genetic finding did not want more children because of risking to pass the pathogenic variant on to offspring

**Table 4:** Abstracted open comments on subjective experiences of genetic testing in general (Opportunity to this given at the end of the questionnaire)

Age 46, tested at 36, 4 children before testing, gene *MLH1*	Very afraid of cancer and death, demanded for hysterectomy straight after testing, but was not operated until at age of 45.
Age 37, tested at 27, 1 child before testing and 3 children after testing, gene *MLH1*	No influence on family planning. Genetic finding has caused other difficulties in life. Encourages her children to have genetic testing.
Age 32, tested at 22, 1 child after testing, gene *MLH1*	Considers the uterus and ovaries a risk. Plans to have surgery after menopause.
Age 42, tested at 26, 1 child before testing and 2 children after testing, gene *MLH6*	Genetic finding has caused uncertainty and anxiety. Grateful for surveillance.
Age 52, tested at 35, 2 children before testing and 1 after testing, gene *MLH1*	First reaction was fear and disgust towards upcoming surveillance procedures. Later grateful for information and her children’s possibility for genetic testing.
Age 58, tested at 42, 1 child before testing, gene *MLH1*	Grateful and positive thoughts. Considers herself safe and privileged for surveillance.
Age 42, tested at 27, 2 children after testing, gene *MLH1*	Grateful for supportive professionals. Tells that surveillance appointments were nor provided automatically at regional hospital, had to insist them.
Age 34, tested at 27, 1 child before testing and 1 after testing, gene *MLH1*	Had depression for 6 months after genetic finding. Other reasons influenced as well. Considers results reported to her in an unfriendly and negative manner. Felt that prophylactic removal of gynecological organs deteriorates self-esteem.
Age 41, tested at 33, 1 child before testing and 3 children after testing, gene *MLH1*	Grateful for testing and surveillance. Worried for her children.
Age 39, tested at 21, 2 children after testing, gene *MLH1*	Genetic finding had negative influence on feminine self-image.
Age 30, tested at 23, 2 children after testing, gene *MSH2*	Was missing peer support, then got it from her own sister after her testing. Grateful for surveillance. Worried for her children.
Age 36, tested at 27, 1 child after testing, gene *MLH1*	Feels safe and does not have worries. Grateful for surveillance.

## Discussion

This descriptive study reveals the subjective views of carriers of *path_MMR* on the influence of germline testing to their important life decisions. Inherited cancer syndromes such as LS do not affect only the individual carrying the germline variant, but also the *path_MMR* carriers’ children have a 50% chance of inheriting the cancer predisposing gene. It is therefore likely that inherited MMR gene variants may affect individual’s decision-making regarding family planning, or relationships in general. Since there were no PMS2 pathogenic variants (PV) in the study population, we were unable to comment on women’s perceptions of being a PMS2 PV carrier.

There is a paucity of data concerning family planning among individuals with inherited cancer syndromes. Even though inherited gene variants conferring gynecological cancer risks do not have impact on fertility as such [17, 18], some reports have implicated that germline testing results have impact on reproductive decisions [8, 9]. Some individuals with pathogenic MMR variants have even been reported to consider prenatal genetic testing and consider it ethical [8, 19]. Therefore, it is very important to collect LS carriers’ subjective views and experiences of genetic testing on their reproductive decisions in order to guide and help clinicians in counselling.According to our results, testing positive for a LS-causing variant appears not to have significant impact on family planning or negative influence on relationships among Finnish women. Only a minority of responders reported any influence or negative impact. Parity of the carriers of *path_MMR* in this study was 2.1, which, to our surprise, was even higher than that of Finnish women in general. The mean parity among Finnish women was 1,8 in 2012 (data from Statistics Finland, stat.fi). The educational background may not explain this, since the education level of the study population does not differ significantly from the general Finnish population.

The number of sterilizations and induced abortions was exactly the same before and after germline testing possibly implying that the fear of passing the pathogenic variant forward is not a major determinant of reproductive decisions. However, in open comments some reported worry, deteriorating of feminine self and body image and fear of having more children. Due to the inclusion criteria, study subjects were relatively young, both at the time of germline testing and at the time of study. However, the mean time interval between testing and present study was 13 years, thus we can assume that these women have been adapted to being carriers of *path_MMR* and not in the initial phase of accepting it. Study subjects were all in surveillance phase and contemporarily considered to have increased risk of endometrial cancer compared to general population.

As in our earlier study [14], women with LS consider the surveillance and the information given by medical professionals of high-quality and very beneficial. According to these women’s subjective opinion, the role of adequate information can be considered very significant in avoiding possible negative psychological impact associated with carrying a *path_MMR*. These variant carriers’ views highlight the experienced impact of regular surveillance on managing the psychological side-effects associated with positive germline testing. This aspect can be underestimated in gynecological surveillance trials, where survival benefit is usually considered as the primary endpoint. Moreover, according to our results, there is a subjective variation how an individual carrier of *path_MMR* experiences the impact of surveillance on her psychosocial wellbeing and it probably should be taken into account in an effort of tailoring the carriers’ counselling and management.

In Finland, women in LS families mainly seem to have adequate knowledge of gynecological cancer screening and they are aware of their entitlement to participate in it. In addition to the clinical specialists, LSRFi offers support and information for the carriers of *path_MMR*. In the present study, all responding women reported attending the gynecological surveillance regularly. None of the subjects in our study implicated not having known about the surveillance. However, concerning the present national guidelines, information of practice, benefits and impact of the gynecological counselling and RRS could probably be improved. Even some false perceptions of the screening were present among the answers. One woman also reported she had to ask for surveillance as it was not provided automatically.

The present study had some limitations. The study population was relatively small and the response rate was low (44.3%). This is possibly due to several study questionnaires that were sent to these women as a part of the larger LS study entity. Some women returned empty questionnaires, implicating in a note that they do not want to be reminded of their cancer predisposition and give additional thought to their genetic risk as they already have to attend the surveillance. Majority of the subjects in the present study were middle-aged or younger at the time of study. Therefore, they were probably in a relatively busy phase of their life and this could partly explain the somewhat low response rate. Data in the present study was self-reported, including the surveillance behavior, but the main goal of the present study was to highlight the true subjective, personal experiences of the carriers of *path_MMR*. Moreover, earlier studies have supported the validity of self-reported information [20]. The strength of the present study is the inclusion of study subjects that are verified carriers of germline MMR gene variant identified from the LSRFi and access to their medical data was used for verification of parity data, time of germline testing etc. It can be also considered a strength that the study subjects were not from a single center but represented Finnish women with LS from various parts of Finland.

## Conclusions

In conclusion, testing positive for a germline variant in their fertile age does not seem to have a significant negative impact on women’s reproductive decisions among the Finnish women with *path_MMR MLH1, MSH2 or MSH6*. The positive germline testing does not seem to confer a negative impact on intimate relationships or on feminine self and body image. Almost all women responding in this study experienced regular surveillance beneficial. The results of the present study can be considered of valuable addition to the counselling of women with LS after germline testing and enables clinicians to share reassuring peer-derived data of reproductive issues to women carrying the *path_MMR*. In addition to preventing gynecological cancer, counselling and caring by specialists after germline testing seems to decrease concerns about variant carriers’ future life. Supportive and empathic attitude of the professionals seems to be a significant factor in avoiding anxiety and fears of the carriers of *path_MMR*. Similar conclusions have been presented in earlier studies on carriers of cancer-related genetic variants [12, 13].

## Data Availability

The datasets used and/or analyzed during the current study are available from the corresponding author on reasonable request.
